# Influence of 25-Hydroxy-Vitamin D Insufficiency on Maximal Aerobic Power in Elite Indoor Athletes: A Cross-Sectional Study

**DOI:** 10.1186/s40798-021-00363-1

**Published:** 2021-10-14

**Authors:** Astrid Most, Oliver Dörr, Holger Nef, Christian Hamm, Timm Bauer, Pascal Bauer

**Affiliations:** 1 Department of Cardiology and Angiology, Justus- Liebig- University Giessen, Klinikstrasse 33, 35392 Giessen, Germany; 2 Kerckhoff Heart and Thorax Center, Bad Nauheim, Germany; 3 Department of Cardiology, Internal Intensive Care, General Internal Medicine, Sana Klinikum, Offenbach, Germany

**Keywords:** 25-OH vitamin D concentration, Indoor athletes, Maximal aerobic power, Cycling ergometer test

## Abstract

**Background:**

The impact of vitamin D on musculoskeletal health is well-established, although its influence on physical performance is unclear. Therefore, we conducted this study to evaluate the impact of 25-hydroxy-vitamin D (25-OH vitamin D) concentrations with maximal aerobic power of professional indoor athletes.

**Results:**

A total of 112 male professional athletes were included in this cross-sectional study, consisting of 88 handball and 24 ice hockey players. The maximal aerobic power was assessed with a standardized cycling ergometer test. Athletes were assigned to two groups according to their 25-OH vitamin D status: insufficient (< 30 ng/mL) and sufficient (≥ 30 ng/mL). Thirty-four players (30.4%) displayed insufficient (21.9 ± 5.9 ng/mL) and 78 (69.6%) sufficient 25-OH vitamin D concentrations (41.6 ± 8.6 ng/mL). Athletes with sufficient levels achieved a higher maximal aerobic power (3.9 ± 0.9 vs. 3.5 ± 0.8 W/kg, *p* = 0.03) compared to those with insufficient levels.

**Conclusions:**

There is a high prevalence of 25-OH vitamin D insufficiency in professional indoor athletes, even in summer. Insufficient 25-OH vitamin D concentrations were associated with lower maximal aerobic power in male professional indoor athletes. Further, the 25-OH vitamin D concentration was identified as the only independent predictor of maximal aerobic power in these athletes, highlighting the impact of 25-OH vitamin D on physical performance. Therefore, 25-OH vitamin D concentrations of ≥ 30 ng/mL should be maintained to ensure optimal physical performance in these athletes.

**Supplementary Information:**

The online version contains supplementary material available at 10.1186/s40798-021-00363-1.

## Key Points

- 25-OH vitamin D insufficiency is associated with a lower maximal aerobic power of professional indoor athletes.
- 25- OH vitamin D concentrations of ≥30 ng/mL should be maintained to ensure optimal physical performance.
- Athletes involved in indoor sports should be evaluated regularly to avoid 25-OH vitamin D insufficiency.

## Background

The importance of vitamin D for a range of physiological processes is uncontested. 25-hydroxy-vitamin D (25-OH vitamin D) plays a key role in calcium homeostasis, bone metabolism, and muscle strength [1]. 25-OH vitamin D deficiency causes osteopenia and osteoporosis, muscle weakness, and increases the risk of falls and fractures. Furthermore, 25-OH vitamin D is thought to have a cardioprotective influence encompassing anti-atherosclerotic, anti-inflammatory, and direct effects at the myocardium [2]. Hence, vitamin D deficiency has been associated with an increased risk of cardiovascular diseases and mortality [2].

Exposure to UV radiation in sunlight is required for synthesis of vitamin D in the skin; this route obtains about 90% of the required amount of vitamin D. Latitude, season of the year, time of day, age, and skin melanin content are important factors that influence vitamin D production [3]. The prevalence of vitamin D deficiency is increasing in the industrialized world due to a lack of sun exposure, dietary differences, clothing, lifestyle (sedentary lifestyle with less outdoor activity), obesity, and the use of sunscreen when outdoors [3–5]. Diet constitutes only a small source of vitamin D [6]. Given the low amounts of vitamin D in the typical western nutrition, a vitamin D-enriched diet seems to be ineffective to prevent vitamin D insufficiency [6].

Most of the vitamin D effects are mediated through the vitamin D receptor, which is expressed in many tissues including parathyroid gland, kidney, intestine, ovarian tissue, in the human endometrium and bones [7, 8]. Regardless of whether it is produced by the skin through ultraviolet (UV) B radiation or whether the source is gastrointestinal uptake, vitamin D is hydroxylated to 25-hydroxy vitamin D (abbreviated 25-OH vitamin D) in the liver by the enzyme vitamin D 25-hydroxylase. 25-OH vitamin D, the major circulating metabolite, is converted to its hormonally active metabolite form, 1,25-dihydroxy-vitamin D (also abbreviated as 1,25(OH)2D). This metabolic step occurs mainly in the kidney tubules. The enzyme 1α-hydroxylase, a member of the family of mitochondrial cytochrome P450 enzymes, is required for conversion and is controlled by parathyroid hormone [5, 9, 10].

The classification of 25-OH vitamin D concentrations as deficient, insufficient, or normal is still controversial, with recommendations for the general population being inconsistent [11–13]. The main circulating metabolite, 25-OH vitamin D, is currently viewed as the parameter that best reflects vitamin D status in athletes and the general population [2, 14–17]. The European guidelines recommend the optimal target 25-OH vitamin D concentration in a range of 30–50 ng/mL (75–125 nmol/L) [15], whereas the Endocrine Society considers serum 25-OH vitamin D concentrations of 40–60 ng/mL (100–150 nmol/L) as optimal levels [11]. Because 25-OH vitamin D concentrations of > 30 ng/mL were shown to minimize bone mineralization defects and thus to maintain skeletal health [18], most studies determined > 30 ng/mL as threshold for sufficient levels [11, 19].

Several recent studies focused on the vitamin D status of athletes [20, 21]. The prevalance of vitamin D insufficiency in athletes has been reported to be between 42 and 83%, which is comparable to that of the general population [3]. Farrokhyar et al. specified in their meta-analysis that 25-OH vitamin D deficiency is more common at higher latitudes, in winter and in early spring, and for indoor sport activities [20].

Sufficient 25-OH vitamin D concentration (> 30 ng/mL) was associated with a lower risk for muscle and bone injuries in elite ballet dancers [1] and stress fractures in high-risk college athletes [22]. Recently, a positive influence of vitamin D on increasing muscle protein synthesis via vitamin D receptors has been suggested [2]. Hence, some researchers even regard 25-OH vitamin D > 50 ng/mL as necessary for professional athletes to ensure optimal athletic performance [23].

Beneficial effects of higher 25-OH vitamin D levels to improve muscle strength in elite athletes were reported [24]. Zhang et al. demonstrated that vitamin D supplementation positively affected lower limb muscle strength in athletes, but not upper limb muscle strength or muscle power [25]. Rockwell et al. pointed out that vitamin D supplementation is an efficacious strategy to maintain 25-OH vitamin D during the fall season training and to enhance some aspects of strength/power and fat-free mass in swimmers [26].

In addition, one study found positive correlations of 25-OH vitamin D status with both muscle strength and VO2_max_ in professional soccer players [27]. In contrast, other researchers found no correlation of 25-OH vitamin D concentration with VO2_max_ [28].

The aim of our cross-sectional study was to assess the relationship between 25-OH vitamin D concentrations and maximal aerobic power, assessed with a standardized exhaustive cycling exercise test, in a homogenous cohort of male professional indoor athletes, referred for routine pre-season clinical examination. We hypothesized that athletes with vitamin D insufficiency would achieve a lower peak performance compared to those with sufficient concentrations.

## Methods

### Study Cohort

This cross-sectional study was carried out in Giessen, Germany, near 50° N latitude during a routine pre-season medical monitoring program after a six-week free interval in terms of competition and team training in July of the years 2015 to 2017. One hundred twelve healthy, injury-free male athletes, consisting of 88 professional handball and 24 ice hockey players, were included. All athletes were Caucasians; none of them were regular sunbed users, took vitamin supplements or showed signs of hypovitaminosis. The youngest included athlete was 18 and the oldest 38 years old.

Medical history, nutrient supplementation, age, weight, height, and body mass index were documented. Professional training duration (years) and training amount per week (hours) were recorded. Furthermore, a physical examination was carried out. 25-OH vitamin D, calcium, and parathyroid hormone concentrations were measured in venous blood samples.

Blood samples were drawn from an antecubital vein in a sitting position. Blood samples for plasma analyses were collected into two 7.5-mL S-Monovette® tubes (Sarstedt AG & Co. KG, Germany), one containing lithium heparin. An additional 2.7-mL sample, with dipotassium ethylene diamine tetra-acetic acid (K2EDTA) as anticoagulant, was acquired (Sarstedt AG & Co. KG, Germany). Within 30 min of blood sampling, automated analysis was performed in the laboratory of the university hospital Giessen. Serum 25-OH vitamin D concentrations were determined with a Liaison diagnostic system (DiaSorin, Stillwater, MN, USA) by chemiluminescent immunoassay (CLIA). The range of detection is 4–150 ng/mL with a precision of 5.0% CV and an accuracy SD of 1.2. Parathyroid hormone was analyzed using an electrochemiluminescent immunoassay (Elecsys PTH (1-84)^®^, Roche Diagnostics, Germany), which measures the circulating active parathyroid hormone. The range of detection is 5.5–2300 pg/mL with a precision range of 2.5% to 3.4% CV. Furthermore, calcium levels, a complete blood cell count and a basic metabolic panel including electrolytes, were assessed and analyzed by a Modular Analytics E 170 module (Roche Diagnostics, Mannheim, Germany).

Based on recently published data and guideline recommendations, the following threshold ranges for 25-OH vitamin D were used: 25-OH vitamin D concentrations of < 30 ng/mL were determined as insufficient, and ≥ 30 ng/mL were regarded as sufficient concentrations [5]. To ensure comparability with other studies, we additionally divided the athletes into four subgroups, according to their respective 25-OH vitamin D concentrations: < 20 ng/mL, ≥ 20– < 30 ng/mL, ≥ 30– < 50 ng/mL and ≥ 50 ng/mL.

Our local ethics committee approved the study protocol (ethics approval number AZ 205/15). Each athlete gave written informed consent. This study was performed in accordance with the standard of ethics outlined in the Declaration of Helsinki [29].

### Exercise Testing

The exercise test was performed between 12:00 pm and 02:00 pm and was scheduled after a 6-week competition-free interval. Athletes did their last training session 36 h prior to the exercise test. The last allowed food intake was up to 3 h before the investigation. There were no restrictions on fluid intake apart from alcohol consumption, which was prohibited the two days prior to commencement of the study. The day before the examination athletes had to abstain from physical exertion.

A progressive, maximal-load cycling ergometer test with concurrent brachial blood pressure measurement and ECG recording was performed (Schiller AG®, Obfelden, Switzerland). The exercise test protocol started with a load level of 100 Watt (W) after a 2-min warm-up period at 50 W. Loads were increased by 50 W every 2 min until exhaustion, which was defined as the participants’ inability to maintain the load for 2 min. The load was then decreased to 25 W for 3 min of active recovery followed by a 2-min cool-down period at rest. The test concluded with a final ECG recording and a brachial blood pressure measurement. We assessed the maximal aerobic power of the athletes, maximum heart rate, heart rate at rest and after the exercise test, and systolic and diastolic brachial blood pressure at rest, during, and after exercise.

### Sun Exposure

The sun exposure questionnaire concerning individual daily sun exposure during the two weeks prior to the examination had been validated for healthy Caucasians [30]. There were three choices for the amount of time spent outdoors each day, and the answers were scored using a point system (0 points for < 5 min, 1 point for 5–30 min, and 2 points for > 30 min). Four choices of clothing or skin exposure while outdoors were assigned points (1 point for face and hands only; 2 points for face, hands and arms; 3 points for face, hands and legs; and 4 points for bathing suit). A total score to estimate their mean weekly sun exposure resulting from the answers was then calculated. The sum of the daily products of time outdoors and skin exposure defined the score for one day, with a minimum score of “0” (lowest amount of time spent outdoors and lowest amount of skin exposed) and a maximum score of “8” (outdoors for more than 30 min in a bathing suit every day). All seven-day sun exposure scores were summed to give the weekly sun exposure score (minimum = 0, maximum = 56).

### Statistical Analysis

Results are presented as means ± standard deviation (SD) for normally distributed data. After testing for normality of the distribution, data were evaluated using the unpaired Student’s t test or the Mann–Whitney U test, as appropriate. The differences between the independent groups were analyzed using the Mann–Whitney U test and ANOVA.

Bivariate relations were analyzed using Spearman correlation coefficient. Pearson’s correlation was used to determine linear correlations between anthropometric parameters, training data, 25-OH vitamin D, and maximal aerobic power, assessed by cycle ergometer and displayed as W/kg.

We performed multiple regression analyses to explore possible linear associations across the anthropometric parameters and training data with the maximal aerobic power, assessed as W/kg. We therefore determined different statistical models. In model 1, we used 25-OH vitamin D, age, weight and height as predictors of the regression model, and the maximal aerobic power as continuous dependent variable. Further, in model 2, we used age, weight, height, 25-OH vitamin D, parathyroid hormone, training per week and training history as predictors of the regression model and, again, the maximal aerobic power as continuous dependent variable. Statistical significance was set at *p* < 0.05 (two-tailed) for all measurements. All statistical analyses were performed using the statistical software SPSS 25.0 for Mac (Statistical Package for the Social Sciences, Chicago, IL, USA).

## Results

The 112 male professional indoor athletes (88 handball players, 24 ice hockey players) had participated in professional training for 9.9 ± 5.2 years with a mean training time of 16.9 ± 3.5 h per week. They were 26.1 ± 5.2 years old (between 18 to 38 years), 189.6 ± 7.4 cm tall, and the body mass index (BMI) was 25.8 ± 1.8 kg/m^2^. Detailed data are given in Tables 1 and 2.

**Table 1:** Characteristics of all 112 athletes

Characteristics	Minimum	Maximum	Mean ± SD
Age (years)	18	38	26.1 ± 5.2
Weight (kg)	64.4	123.0	92.9 ± 10.7
Height (cm)	169.0	204.0	189.6 ± 7.4
Body mass index (kg/m^2^)	22.0	33.0	25.8 ± 1.8
Professional training (years)	1.0	22.0	9.9 ± 5.2
Training time per week (hours)	9.3	23.3	16.9 ± 3.5

Values are reported as means ± standard deviation (SD), maximum and minimum

**Table 2:** Characteristics of athletes with insufficient (< 30 ng/mL) and sufficient (≥ 30 ng/mL) serum 25-OH vitamin D concentrations

Characteristics	Insufficient	Sufficient	*p* value
	(< 30 ng/mL)	(≥ 30 ng/mL)	
	*n* = 34	*n* = 78	
	Mean ± SD	Mean ± SD	
Serum 25-OH vitamin D (ng/mL)	21.9 ± 5.9	41.6 ± 8.6	**< 0.001**
Age (years)	27.4 ± 5.9	25.6 ± 4.8	0.13
Weight (kg)	93.5 ± 11.1	92.6 ± 10.6	0.66
Height (cm)	190.0 ± 8.1	189.4 ± 7.2	0.71
Body mass index (kg/m^2^)	25.8 ± 1.5	25.8 ± 1.9	0.81
Training time per week (h)	16.2 ± 3.7	17.2 ± 3.4	0.28
Professional training (years)	10.23 ± 5,8	9.82 ± 5	0.77
Parathyroid hormone (ng/mL)	40.5 ± 16.0	32.0 ± 18.2	**0.02**
Calcium (mmol/l)	2.3 ± 0.14	2.4 ± 0.1	0.14
Hemoglobin (g/dl)	14.8 ± 1.0	15.0 ± 0.9	0.40
Hematocrit (%)	42.5 ± 2.4	42.6 ± 2.3	0.86
Ferritin (ng/ml)	108.0 ± 52.5	139.5 ± 73.3	0.05
Magnesium (mmol/l)	0.82 ± 0.07	0.82 ± 0.05	0.98
Maximal aerobic power (w/kg)	3.5 ± 0.8	3.9 ± 0.9	**0.03**
Maximal workload (watt max.)	330.9 ± 85.7	363.1 ± 94.0	0.08
Heart rate at rest (bpm)	58.5 ± 8.6	58.8 ± 10.9	1.00
Maximum heart rate (bpm)	175 ± 16.4	176.9 ± 10.0	0.62
Systolic blood pressure at rest (mmHg)	119.1 ± 11.4	120.1 ± 8.3	0.58
Diastolic blood pressure at rest (mmHg)	80.2 ± 7.2	74.7 ± 6.9	**0.004**
Max. systolic blood pressure (mmHg)	186.3 ± 18.7	193.7 ± 21.2	0.14
Max. diastolic blood pressure (mmHg)	86.8 ± 6.7	87.3 ± 9.9	0.79
LVEF (%; echocardiography)	66.5 ± 4.7	65.5 ± 4.6	0.40
Stroke volume (ml, echocardiography)	84.0 ± 17.5	87.5 ± 17.4	0.51

Values are specified as means ± standard deviation (SD)

Significant difference (*p* < 0.05)

W = watt, max. = maximum, bpm = beats per minute, LVEF = left ventricular ejection fraction

The mean serum concentration of 25-OH vitamin D was 36.4 ± 12.4 ng/mL for all athletes with a maximum value of 70.7 ng/mL and a minimum value of 8.4 ng/mL. Thirty-four athletes (30.4%) were found to be 25-OH vitamin D insufficient (21.9 ± 5.9 ng/mL) and 78 players (69.6%) displayed sufficient concentrations (41.6 ± 8.6 ng/mL).

The mean calcium level was 2.4 ± 0.1 mmol/l for the entire cohort (insufficient 25-OH vitamin D group 2.3 ± 0.14 mmol/l versus sufficient group 2.4 ± 0.1 mmol/l) and the mean parathyroid hormone concentration was 34.6 ± 18.0 pg/mL. Lower parathyroid hormone concentrations were observed in athletes with sufficient 25-OH vitamin D blood levels than in those with insufficiency (32.0 ± 18.2 vs. 40.5 ± 16.0 ng/mL; *p* = 0.02). Other blood parameters such as hemoglobin, hematocrit, ferritin and magnesium did not differ significantly between the two groups. Athletes with sufficient 25-OH vitamin D concentrations achieved a significantly (*p* =  < 0.05) higher performance level than athletes with insufficient concentrations (3.9 ± 0.9 versus 3.5 ± 0.8 W/kg).

There were no significant differences regarding age, height, weight, body mass index, sun exposure points, or training history between athletes in the sufficient versus insufficient groups. Correspondingly, heart rate at rest, maximum heart rate, systolic blood pressure at rest, maximum systolic blood pressure, maximum diastolic blood pressure, left ventricular ejection fraction (LVEF) and stroke volume did not differ significantly between the two groups. In contrast, diastolic blood pressure at rest was significantly lower in the sufficient group (74.7 ± 6.9; versus 80.2 ± 7.2, *p* = 0.004). These results are shown in Table 2.

Finally, we divided the athletes into additional groups of 25-OH vitamin D concentrations as described above (< 20 ng/mL, ≥ 20– < 30 ng/mL, ≥ 30– < 50 ng/mL and ≥ 50 ng/mL) to investigate between-group differences (Table 3 and Additional file 1).

**Table 3:** Characteristics of athletes according to the different serum 25-OH vitamin D concentrations (four subgroups)

	Serum 25- OH vitamin D concentrations			
	< 20 ng/mL	≥ 20– < 30 ng/mL	≥ 30– < 50 ng/mL	≥ 50 ng/mL
	(*n* = 10)	(*n* = 24)	(*n* = 61)	(*n* = 17)
	Mean ± SD	Mean ± SD	Mean ± SD	Mean ± SD
Age (years)	26.3 ± 7	27.8 ± 5.4	25.5 ± 5.1	25.6 ± 3.9
Height (cm)	192 ± 7	189 ± 8.4	189 ± 7.5	190 ± 6
Weight (kg)	93.8 ± 9.8	93.5 ± 11.8	91.5 ± 10.2	96.5 ± 11.1
BMI (kg/m^2^)	25.8 ± 1.36	26 ± 1.56	25.2 ± 1.72	26.5 ± 2.33
PTH (ng/mL)	48.8 ± 10.2	37.3 ± 17	34.1 ± 19	**24.3 ± 12.8 **^**a**^
Calcium (mmol/L)	2.36 ± 0.1	2.3 ± 0.1	2.39 ± 0.08	2.4 ± 0.07
Serum 25-OH vitamin D (ng/mL)	14.6 ± 3.9	**25.8 ± 2.7**^**a**^	**38.6 ± 5.2**^**a**^**/**^**b**^	**56.5 ± 5.2**^**a**^**/**^**b**^**/**^**c**^
Maximal workload (W)	295 ± 48	340 ± 74	**351 ± 58**^**a**^	**406 ± 30**^**a**^**/**^**b**^
Maximal aerobic power (W/kg)	3.18 ± 0.64	3.59 ± 0.71	**3.85 ± 0.46**^**a**^	**4.24 ± 0.36**^**a**^**/**^**b**^
Maximum heart rate (bpm)	175 ± 15	175 ± 17	176 ± 10	177 ± 10
Maximum SBP (mmHg)	183 ± 19	187 ± 19	193 ± 19.5	195 ± 26
Maximum DBP (mmHg)	86.7 ± 5.6	86.8 ± 7.4	86.8 ± 9.7	88.8 ± 11
Resting heart rate (bpm)	59.2 ± 10	58.2 ± 8	58.2 ± 10	59.3 ± 13.9
Resting SBP (mmHg)	120 ± 12	119 ± 12	119 ± 7.4	126 ± 9
Resting DBP (mmHg)	81.6 ± 5	79.4 ± 10.3	**74.9 ± 6.2**^**a**^	**74.1 ± 8.9**^**a**^**/**^**b**^
SEP (points)	73.2 ± 23.8	75.5 ± 20.1	78.6 ± 15	80.6 ± 28.5

See Additional file 1 for specific *p* values. Bold text signifies significant differences. Values are given as means ± standard deviation (SD) and as median with interquartile ranges (IQR)

BMI = body mass index; PTH = parathyroid hormone; SE* p* = sun exposure points (measured over the two weeks prior to examination; maximum 112 points)

^**a**^Significant difference vs. concentrations of < 20 ng/mL (*p* < 0.05);

^**b**^Significant difference vs. concentrations of ≥ 20- < 30 ng/mL (*p* < 0.05);

^**c**^Significant difference vs. concentrations of ≥ 30- < 50 ng/mL (*p* < 0.05)

Athletes with 25-OH vitamin D concentrations ≥ 50 ng/mL displayed significantly lower parathyroid hormone concentrations than participants with 25-OH vitamin D concentrations < 20 ng/mL (*p* = 0.004). Additionally, they had a significantly lower diastolic blood pressure at rest (*p* = 0.039) than athletes with concentrations of ≥ 20– < 30 ng/mL. Participants with 25-OH vitamin D concentrations ≥ 30 ng/mL (≥ 30- < 50 ng/mL and ≥ 50 ng/mL) had a significantly lower (*p* < 0.05) diastolic blood pressure at rest (74.9 ± 6.2 and 74.1 ± 8.9 mmHg, respectively) than athletes with concentrations < 20 ng/mL (81.6 ± 5 mmHg).

In addition, athletes with 25-OH vitamin D concentrations ≥ 30 ng/mL (≥ 30– < 50 ng/mL and ≥ 50 ng/mL) achieved a significantly (*p* < 0.05) higher maximum workload and maximal aerobic power than athletes with 25-OH vitamin concentrations < 20 ng/mL. This significant difference (*p* = 0.049) was also observed when comparing the groups with concentrations of ≥ 50 ng/mL (406 ± 30 W, 4.24 ± 0.36 W/kg) with the group of ≥ 20– < 30 ng/mL (340 ± 74 W, 3.59 ± 0.71 W/kg).

There were no significant correlations between 25-OH vitamin D concentrations, age (*r* =  − 0.084, *p* = 0.38), body height (*r* =  − 0.67, *p* = 0.48), weight (*r* =  − 0.009, *p* = 0.92), BMI (*r* = 0.065, *p* = 0.49), professional training time (*r* =  − 0.214, *p* = 0.42) and training time per week (*r* = 0.116, *p* = 0.30). Though, we found a positive correlation between 25-OH vitamin D concentrations and maximal aerobic power (*r* = 0.555, *p* = 0.03).

We performed multiple regression analyses to explore possible linear associations across the anthropometric parameters and training data with the maximal aerobic power, assessed as W/kg. We therefore determined different statistical models.

In model 1, we used 25-OH vitamin D, age (*β* = 0.009, *p* = 0.925), weight (*β* = − 0.181, *p* = 0.692) and height (*β* = 0.123, *p* = 0.834) as independent predictors of the maximal aerobic power. This model was not able to predict maximal aerobic power (*p* = 0.054, *r*^2^ = 0.042, *F* = 2.631, Durbin–Watson 2.132). With the Durbin–Watson statistic indicating no collinearity between the predictors, the results suggest that only 25-OH vitamin D (*β* = 0.227, *p* = 0.017) significantly predicts maximal aerobic power.

An extended model that included as independent predictors parathyroid hormone (*β* = 0.059, *p* = 0.644), training per week (*β* = 0.083, *p* = 0.510) and training history (*β* = − 0.186, *p* = 0.686) in addition to 25-OH vitamin D (*β* = 0.296, *p* = 0.018), age (*β* = 0.181, *p* = 0.692), weight (*β* = 0.169, *p* = 0.171) and height (*β* = 0.175, *p* = 0.356), did not change the result. The extended model was not able to predict maximal aerobic power (*p* = 0.276, *r*^2^ = 0.020, *F* = 1.282, Durbin–Watson 2.209). Again, 25-OH vitamin D remained as the only significant predictor of maximal aerobic power.

**Fig. 1:**
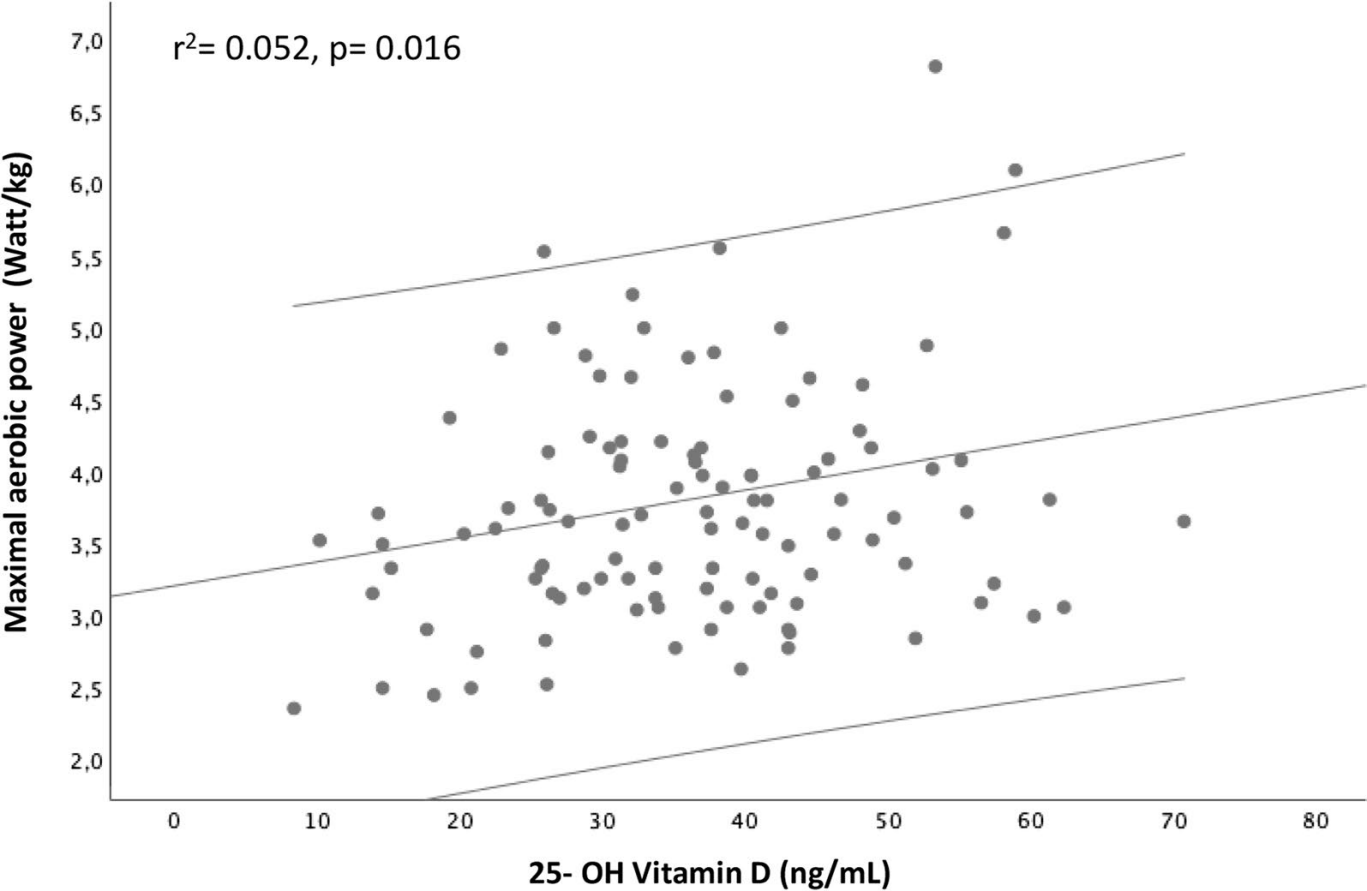
Maximal aerobic power as a function of 25-OH vitamin D

In single linear regression analysis with 25-OH vitamin D concentration as the only predictor of maximal aerobic power, it was found that 25-OH vitamin D concentration significantly predicted maximal aerobic power (*r*^2^ = 0.052, *p* = 0.016, *F* (1.112) = 5.986, Durbin–Watson statistic 2.188). The regression result is shown in Fig. 1.

## Discussion

This cross-sectional study examined the relationship between 25-OH vitamin D concentrations and maximal aerobic power in male professional indoor athletes.

Our main finding was that athletes with sufficient 25-OH vitamin D concentrations displayed a higher maximal aerobic power compared with athletes with insufficient 25-OH vitamin D concentrations. Further, 25-OH vitamin D concentrations predicted maximal aerobic power in multivariate regression analyses.

We report a high prevalence of 25-OH vitamin D insufficiency (30.4%) even in summer, when 25-OH vitamin D levels are expected to peak. Our data were collected following a six-week competition-free interval after summer holidays. Consequently, we measured a high sun exposure score without differences between athletes with sufficient versus those with insufficient 25-OH vitamin D values. Several studies have documented seasonal differences in 25-OH vitamin D concentrations in athletes [3, 16] with indoor athletes being at higher risk for developing 25-OH vitamin D insufficiency [2, 24]. This unmasks the problem inherent in using this validated sun exposure score questionnaire, which only evaluates the sun exposure during the preceding 2 weeks. In addition, the widespread use of sunscreen is not considered, which constitutes a major limitation of this measurement method.

Other studies found a higher prevalence of 25-OH vitamin D insufficiency in athletes [21, 31] with 79.3% of the players of the National Basketball Association (NBA) being classified as either vitamin D deficient (< 20 ng/mL) or insufficient (20–32 ng/mL) [31]. These differences might be explained with the cohorts investigated, as a higher percentage of these basketball players were African Americans, and dark skin constitutes a well-known risk for vitamin D deficiency.

The main finding of our study is the significant correlation of 25-OH vitamin D concentrations with maximal aerobic power. Athletes with insufficient 25-OH vitamin D concentrations displayed a lower maximal aerobic power than those with sufficient concentrations.

The results of studies investigating the associations of 25-OH vitamin D with exercise performance in athletes have been inconclusive [24, 27, 32, 33]. In general, given the inconsistent definitions of insufficient 25-OH vitamin D concentrations in the literature, a comparison between studies is challenging. Moreover, different exercise testing methods and determination of performance levels limit comparisons. Other important factors to consider when reviewing recent literature is the sports type investigated, the baseline individual training profile, age and sex, especially in athletic populations. Professional athletes typically have minimal margins for improvement due to their exceptionally high training status. This might explain the comparatively small, though significant, percentage of maximal performance variance explained by 25-OH vitamin D in our athletes. The observation by Ardestani et al. that the effect of 25-OH vitamin D is the greatest in individuals with low levels of physical activity supports this contention [34].

It was suggested that vitamin D may improve muscle mass and strength. Further positive effects of higher vitamin D levels on skeletal muscle regeneration and on reparative processes following exercise have been suggested [2]. Hence, 25-OH vitamin D may affect physical performance via direct improvement of muscular function and, in addition, via muscular regeneration processes [2]. Correspondingly, positive associations between serum concentrations of 25-OH vitamin D and skeletal muscle strength in professional judo athletes [24] and elite soccer players were reported [27]. In professional ice-hockey athletes, positive associations of 25-OH vitamin D concentrations with measures of maximal-intensity exercise performance (grip strength, vertical jump performance, and power production during the Wingate Anaerobic Test) were shown [35].

In male recreational athletes with vitamin D insufficiency, a significantly lower submaximal physical performance on a treadmill ergometer was found compared to those with sufficient concentrations [36]. Similar associations were observed in individuals at different stages of life, with focus on both ordinary everyday musculoskeletal tasks and peak athletic performance [37].

Overall, the influence of vitamin D on muscle strength, especially lower muscle strength in professional athletes, was evident in most studies and was physiologically explained by different vitamin D receptor expressions in various muscle groups. Vitamin D affects the number and diameter of type II muscle fibers, which mainly regulate the ability to perform short high-power exercises [38]. These considerations are important in the interpretation of our own results, as we included experienced elite handball and ice- hockey athletes. Competitive team handball and ice-hockey are both classified as high-intensity mixed sports which impose high loads on the cardiovascular system. They are characterized by repetitive bouts of high-intensity activities interspersed with brief recovery periods. Players need the ability to perform repeatedly at maximal or near maximal intensities such as sprinting and quick changing of directions throughout the match.

A positive influence of vitamin D on accelerated muscle recovery after intensive exercise has also been suggested. This aspect is of particular relevance to highly trained athletes who are exposed to frequent and repetitive bouts of intensive exercise throughout the week [2]. As muscle biopsies and signaling pathways were not part of our investigation, we are unable to speculate how vitamin D-expedited recovery might have influenced our results.

## Limitations and Strengths of the Study

Several limitations should be considered in interpreting our results. First, we did not have a control group (outdoor athletes or untrained persons), and we assessed the 25-OH vitamin D concentrations and the maximal aerobic power at a single time point in summer. Hence, longitudinal data and information about 25-OH vitamin D concentrations and physical performance in spring, fall and winter are missing. Second, we did not evaluate habitual dietary intake of the athletes to assess nutritional vitamin D uptake. This might have affected our results. Third, self-reported data via questionnaire to obtain information about sun exposure might not be a reasonably accurate method to assess the real impact of natural sunlight on the 25-OH vitamin D concentrations in our athletes. In particular, the lack of information about sunscreen use has to be seen as a major limitation in this setting. Moreover, our study participants were all male Caucasians, so that the results of our study cannot be translated to other professional athletes in general. Another limitation is possibly the cross-sectional design of our study. Cross-sectional studies are observational in nature, analyzing data from individuals of a population at a single point in time. This precludes inferring causality [39].

A notable strength of our investigation is the homogenous cohort of experienced male elite athletes sharing the same age, anthropometry, freedom from cardiovascular disease and being medication naïve. The rigorous assessment of maximal aerobic power, using a standardized exhaustive exercise test, further strengthens our analysis.

## Conclusions

In conclusion, we report a high prevalence of 25-OH vitamin D insufficiency in male professional indoor athletes, even in summer when peak levels are reached. 25-OH vitamin D insufficiency was associated with lower maximal aerobic power, as assessed with a standardized exhaustive cycling ergometer test. Furthermore, the 25-OH vitamin D concentration was identified as the only independent predictor of maximal aerobic power in these athletes, highlighting the impact of 25-OH vitamin D on physical performance.

Therefore, our data support the implementation of monitoring 25-OH vitamin D concentrations in professional indoor athletes throughout the season to avoid insufficient 25-OH vitamin D concentrations.

Whether the threshold of 30 ng/mL 25-OH vitamin D, which was recommended to maintain skeletal health, is also suitable to ensure optimal physical performance in professional athletes, should be addressed in further prospective studies.

## Supplementary Information

**Additional file 1.** p-values for comparison of values for each variable from Table 3 with the different groups.

## Data Availability

The datasets used and/or analyzed during the current study are available from the corresponding author on reasonable request.

## Abbreviations

BMI: Body mass index
ECG: Electrocardiogram
max: Maximal
min: Minutes
MPO: Maximum power output
SD: Standard deviation
VO2_max_: Maximum oxygen uptake
W: Watt
