# Supplementary material for: Influence of 25-Hydroxy-Vitamin D Insufficiency on Maximal Aerobic Power in Elite Indoor Athletes: A Cross-Sectional Study
Source: Sports Med Open. 2021 Oct 14;7:74. doi: 10.1186/s40798-021-00363-1 (PMC8517059; doi:10.1186/s40798-021-00363-1)
Supplement: Supplementary file 1 — Additional file 1. p-values for comparison of values for each variable from Table 3 with the different groups. [file 40798_2021_363_MOESM1_ESM.docx]

**Influence of 25**-**hydroxy**-**vitamin** **D insufficiency on maximal aerobic power in elite indoor athletes- a cross-sectional study**

**Sports Medicine - Open**

**Astrid Most MD^a^, Oliver Dörr MD^a^, Holger Nef MD^a^, Christian Hamm MD^a,b^, Timm Bauer MD^c^, Pascal Bauer MD^a^**

1. **Department of Cardiology and Angiology, Justus- Liebig- University Giessen, Giessen, Germany**
2. **Kerckhoff Heart and Thorax Center, Bad Nauheim, Germany**
3. **Department of Cardiology, Internal Intensive Care, General Internal Medicine, Sana Klinikum, Offenbach, Germany**

**Corresponding author:**

Dr. med. Astrid Most, MD

Department of Cardiology and Angiology

Justus- Liebig- University Giessen

Klinikstrasse 33

35392 Giessen

Germany

Office phone number +49641-985-56702

Fax: +4941-985-42109

e-mail: [astrid.most@innere.med.uni-giessen.de](mailto:astrid.most@innere.med.uni-giessen.de)

**p-values for comparison of values for each variable from Table 3 with the different groups**

Serum 25- OH vitamin D concentrations (ng/mL)

<20 vs. ≥20- <30 <20 vs. ≥30- <50 <20 vs. ≥50 ≥20- <30 vs. ≥50 ≥20- <30 vs. ≥30- <50 ≥30- <50 vs. ≥50

Age 1.0 1.0 0.52 1.0 0.452 1.0

Height 1.0 1.0 1.0 1.0 1.0 1.0

Weight 1.0 1.0 1.0 1.0 1.0 0.54

BMI 1.0 1.0 0.58 1.0 1.0 0.213

PTH 0.57 0.108 0.004* 0.110 1.0 0.235

Calcium 0.87 0.64 0.154 0.35 1.0 1.0

Serum 25-OH vitamin D <0.001* <0.001* <0.001* <0.001* <0.001* <0.001*

Maximal workload 0.80 0.038* 0.014* 0.049* 1.0 0.168

Maximal aerobic power 0.74 0.039* 0.019* 0.041* 1.0 0.654

Max. heart rate 1.0 1.0 1.0 1.0 1.0 1.0

Max. SBP 1.0 1.0 1.0 1.0 1.0 1.0

Max. DBP 1.0 1.0 1.0 1.0 1.0 1.0

Resting heart rate 1.0 1.0 1.0 1.0 1.0 1.0

Resting SBP 1.0 1.0 1.0 0.174 1.0 0.061

Resting DBP 1.0 0.028* 0.014* 0.039* 0.072 1.0

SEP 0.77 0.132 0.086 0.85 0.89 1.0

p-values for comparison of values for each variable from Table 3 with the different groups, according to the respective serum 25-OH vitamin D concentrations

*Significant difference (p<0.05). BMI = body mass index; PTH = parathyroid hormone; SEP = sun exposure points (measured over the two weeks prior to examination;
maximum 112 points).
